# Improving reporting standards in nutrition trials: a collaboration between FENS and the EQUATOR Network

**DOI:** 10.1007/s00394-024-03413-y

**Published:** 2024-05-07

**Authors:** Michael M Schlussel, Jessica Rigutto-Farebrother

**Affiliations:** 1 UK EQUATOR Centre, Centre for Statistics in Medicine, Nuffield Department of Orthopaedics, Rheumatology & Musculoskeletal Sciences, University of Oxford, Oxford, UK; 2 Laboratory of Nutrition and Metabolic Epigenetics, Institute of Food, Nutrition and Health, ETH Zürich, LFV A44, Schmelzbergstrasse 7, Zürich, 8092 Switzerland

Lack of transparency and incomplete description of research methods and findings are known problems in the healthcare literature and can be even more problematic for a complex area of research like nutrition interventions. Reporting guidelines are simple tools that aid researchers in ensuring that the minimum amount of information needed to critically appraise study methods and make sense of the findings is included in publications. However, evidence-based reporting guidelines extensions for studies of nutritional interventions are not available.

As part of the Federation of European Nutrition Societies (FENS) improving standards in the science of nutrition initiative [1], the working group orientated towards improving communication and public trust in nutrition science chose, as part of their mandate, to lead an effort to evaluate and identify key elements of human nutrition intervention trials that should be reported in a standardised way to provide a more robust evidence base. To date, a draft set of recommendations for a nutrition-specific extension to the 25-item CONsolidated Standards Of Randomised Trials (CONSORT) checklist has been proposed, as described in Rigutto-Farebrother et al. (2023) [2] and Weaver et al. (2023) [3], with further peer feedback being obtained through a Delphi survey underway at the time of writing [4].

Concurrently, the Supporting Transparency And Reproducibility in studies of NUTritional interventions (STAR-NUT) [5] working group, hosted within the Enhancing the QUAlity and Transparency Of health Research (EQUATOR) network, designed a research programme following EQUATOR methodology to support transparency and reproducibility for wider nutrition interventions research. STAR-NUT aims to deliver evidence-based developments for the three reporting guidelines that cover the whole nutrition intervention research pipeline from reporting of nutrition trials protocols, randomised controlled trials with nutrition or diet-related interventions, to meta-analyses of such studies. To date, a series of comprehensive literature reviews [6, 7] identifying and informing the main reporting limitations in this area is now complete and submitted for publication at the time of writing. Funding has also been acquired for a consensus meeting that will discuss the format and items to be included in nutrition extensions for the relevant reporting guidelines, i.e., the SPIRIT [8], CONSORT [9] and PRISMA [10] statements.

The results of these two initiatives so far support that developments for reporting guidelines focusing on nutritional interventions are needed and welcome by the nutrition research community. Further, these initiatives are both synergistic and highly complementary. With this letter, we therefore wish to announce that, thanks to meetings between representatives of STAR-NUT and the FENS working group held during the 14th European Conference of Nutrition (FENS 2023), a collaboration was cemented resulting in combined leadership of the next and final steps in the development of consolidated reporting guidance for studies of nutritional interventions ( i.e., “CONSORT-Nut”). The two groups will get together at the aforementioned consensus meeting and based on the evidence gathered with their respective efforts, finalise the recommended reporting items for CONSORT-Nut, summarised in a checklist and set of worked examples for good reporting. Readers wishing to stay updated and/or engage in this process should follow @consort_nut or connect with either of the authors on LinkedIn or by email directly.

Promoting transparency and completeness in reports describing findings from nutrition interventions studies can improve the standards and increase credibility in this area of knowledge. Therefore, by supporting authors to publish articles with better reporting quality, our efforts will benefit those for whom research on nutritional interventions is conducted in the first place: patients and the public.

Financial interests: MMS declares he has no financial interests. JRF declares that she has received reimbursement for conference fees from FENS as part of this initiative. Non-financial interests: MMS declares, on behalf of the STAR-Nut steering group, that all members work directly for or have collaborations with the EQUATOR Network, an international initiative that seeks to improve the reliability and value of published health research literature by promoting transparent and accurate reporting and wider use of robust reporting guidelines. JRF declares, on behalf of the FENS working group, that all members have voluntary collaborations with FENS as part of this initiative. 
